# Evaluation of sleeping problems among caregivers of children that take therapy in the national center for children rehabilitation and treatment during COVID-19 pandemic

**DOI:** 10.1192/j.eurpsy.2021.778

**Published:** 2021-08-13

**Authors:** F. Dobi, B. Zenelaj, E. Kabili, E. Ruci, E. Dokle

**Affiliations:** Child And Adolescent Psychiatry, National Center for Children Rehabilitation and Treatment, Tirana, Albania

**Keywords:** COVID-19, developmental disorder, caregivers, sleeping disorder

## Abstract

**Introduction:**

Compared to the parents of kids with “typical” development the stress level and exhaustion in these parents is higher and more frequent. Furthermore COVID-19 pandemic can increase stress levels especially among people that suffer from mental health disorders. Studies show that these difficult, challenging times have had a negative impact on most families, which have a child with neurodevelopmental disorders.

**Objectives:**

Evaluation of sleeping problems among caregivers of children that take therapy in the National Center for Children Rehabilitation and Treatment (NCCRT) during COVID-19 pandemic

**Methods:**

The study was conducted during a two-month period March-April 2020. The sample involved 110 individuals, relatives, of children that were taking educative and rehabilitation therapy in NCCRT during last year, ambulatory or inpatients. Data were collected by clinical records and phone interviews with children’s caregiver. Instrument we used were: Demographic inventory and Hamilton Anxiety Rating Scale for anxiety symptom evaluation. All data were statistically analyzed through excel.

**Results:**

Most of individual interviewed were parents, 69% of them. 56% of individuals were among 31-45 years old. 28,2% of individuals developed sleeping difficulties and they weren’t able to sleep within 30 minutes after going to bed. 12,7% of them reported to have had difficulties staying awake during driving, eating or other daily activities.

**Conclusions:**

It is necessary the dynamic support with special attention for caregivers whom have sleeping problems. Yet has to be evaluated the connection, if it’s present, between parents with sleeping problems and children progress, for ones that are being supported with development therapy

